# Family in Crisis: Do Halfway Houses Perform Better Than Families with Expressed Emotion toward Patients with Schizophrenia? A Direct Adjusted Comparison

**DOI:** 10.3390/healthcare12030375

**Published:** 2024-02-01

**Authors:** Panagiotis Ferentinos, Stamatina Douki, Vasiliki Yotsidi, Eleni Kourkouni, Dimitra Dragoumi, Nikolaos Smyrnis, Athanasios Douzenis

**Affiliations:** 1 2nd Department of Psychiatry, National and Kapodistrian University of Athens, “Attikon” University General Hospital, 12 462 Athens, Greece; smyrnis@med.uoa.gr (N.S.); thandouz@med.uoa.gr (A.D.); 2 Department of Psychiatry, “Evangelismos” General Hospital, 106 76 Athens, Greece; tan.douki@gmail.com (S.D.); dragoumi@gmail.com (D.D.); 3 Department of Psychology, Panteion University of Social and Political Sciences, 176 71 Athens, Greece; v.yiotsidi@panteion.gr; 4 Center for Clinical Epidemiology and Outcomes Research, 15 451 Athens, Greece; e.kourkouni@cleoresearch.org

**Keywords:** criticism, emotional overinvolvement, expressed emotion, families, halfway houses, schizophrenia, supported housing

## Abstract

Expressed emotion (EE) toward patients with schizophrenia is typically reported to be lower in psychiatric halfway houses than in families. This is the first study directly comparing EE between these settings and investigating the pathways mediating EE differences. We included 40 inpatients in halfway houses and 40 outpatients living with their families and recorded 22 psychiatric nurses’ and 56 parents’ EE, respectively, through Five Minutes Speech Samples. Each inpatient was rated by 2–5 nurses and each outpatient by 1–2 parents. As EE ratings had a multilevel structure, generalized linear mixed models were fitted, adjusting for patient-related confounders and caregiver demographics. Mediatory effects were investigated in multilevel structural equation models. Outpatients were younger, less chronic, and better educated, with higher negative symptoms and perceived criticism than inpatients. Nurses were younger and better educated than parents. Before adjustment, EE rates were equally high across settings. After adjusting for patient-related confounders, emotional overinvolvement was significantly higher in parents. However, after also adjusting for caregiver demographics, only criticism was significantly higher in nurses. Patients’ age, negative symptoms, and perceived criticism and caregivers’ age and sex significantly mediated EE group differences. Our findings highlight pathways underlying EE differences between halfway houses and families and underscore the importance of staff and family psychoeducation.

## 1. Introduction

The family emotional climate has long been recognized as a major predictor of the course of schizophrenia along with stressful life events, reduced compliance with treatment, substance misuse, and poor premorbid adjustment [1,2,3]. The family environment is associated with the emergence of relapses through ‘expressed emotion’ (EE) [4], a construct introduced in the 1950s to describe family members’ affective attitudes and behaviors toward the patient. It refers mainly to criticism (i.e., critical or disapproving comments) and emotional overinvolvement (EOI) (i.e., over-intrusive, over-protective, or self-sacrificing behavior) but also encompasses hostility (i.e., rejection, often highly correlated with criticism) as well as positive aspects not used in scoring EE, that is warmth (i.e., concern and interest for patients, often negatively correlated with criticism and positively with EOI) and positive comments (i.e., praising or approving comments) [5]. Expressed emotion is traditionally assessed with the Camberwell Family Interview (CFI) as well as with the less time-consuming Five Minutes Speech Sample, while several self-report scales have also been used to a lesser extent [6]. Meta-analyses of various prospective studies have documented the negative impact of high EE in family settings, particularly high criticism, on patients’ clinical outcomes [7,8], while high levels of warmth were found to act protectively [9].

Earlier hypotheses about the emergence of EE focused on psychological, i.e., cognitive and emotional, factors, such as caregivers’ personality characteristics [10,11], causal attributions of mental illness [12,13], and locus of control [14]. In particular, high criticism was related to lower openness and flexibility of caregivers, causal attributions of schizophrenia to personal, internal, and controllable factors, and an internal locus of control (i.e., belief in the individual’s own hold of one’s life), while high EOI was related to higher neuroticism, feelings of guilt and self-blame, and causal attributions of disease to universal, external, and uncontrollable factors [15]. More recent research has shown that individuals with schizophrenia [16,17] and, to a lesser extent, their first-degree relatives [18] show stable deficits in various social cognition domains including facial emotion perception, social and non-verbal cue recognition, theory of mind, empathy, and attributional style. These impairments in both patients and their relatives might act in a vicious circle and negatively impact the quality of their communication, thus contributing to the rise and maintenance of EE [19]. However, this hypothesis remains largely understudied [20].

During the last three decades, research on EE in schizophrenia has extended to the staff (usually mental health nurses) of psychosocial rehabilitation services, such as supported housing facilities in the community, a cornerstone of deinstitutionalization [21]. Among them, psychiatric halfway houses (or transitional hostels) provide housing for a limited time frame, probably away from a dysfunctional family, preparing recently hospitalized patients for independent community living and their social reintegration. However, as staff may emotionally invest less in patients than family relatives, high EE in staff–patient studies almost always arises from criticism rather than EOI, which seems less relevant in these settings [22]. Staff EE or its components have also been associated with patients’ outcomes, though weakly, inconsistently, and in much fewer prospective studies [23,24,25].

In cases when the family is deemed to go through a ‘crisis’ or the family climate is ‘toxic’, that is, overly critical or emotionally laden, clinicians often advise patients with schizophrenia to temporarily move to a community residential facility as a means to prevent a relapse or other unfavorable outcomes [26,27]. Rates of high criticism and EOI in supported housing facilities are typically reported to be lower than in families [22]. However, this inference is only based on indirect comparisons between family relatives and staff, since studies including patients from both settings are lacking. Yet, indirect comparisons can suffer from many biases. Measures used for EE, scoring algorithms, as well as data collection procedures followed, may vary significantly among studies. Furthermore, various patient-related (i.e., demographics, clinical parameters, psychopathology, perceived criticism) and rater-related (i.e., family relative or staff) characteristics (i.e., demographics, psychiatric history, caregiver burden) previously associated with EE [15,22] can be distributed unevenly among settings and, hence, act as confounders. Therefore, the question remains: Does supported housing actually perform better than families on EE or its components? And in this case, which factors may explain EE differences across settings?

Consequently, studies directly comparing (i.e., using the same EE measures and scoring algorithms, following the same procedures, and including identical sets of predictors) patients with schizophrenia living in community residential settings and those living in families are highly warranted. This study aimed to directly compare patients with schizophrenia living in halfway houses or with their families on the EE of the caring staff or their parents, respectively, adjusting for patient- and caregiver-related characteristics as confounders, that is, assuming that patient characteristics or both patient and caregiver characteristics were similar across settings. We hypothesized that parents would display higher EOI than nurses, but levels of criticism would not be different. Finally, we also aimed to investigate the pathways underlying EE differences between the two settings. That is, which patient or caregiver characteristics, when different between the two settings, would make EE also differ across settings?

## 2. Materials and Methods

### 2.1. Participants and Settings

A convenience sample of a total of 80 individuals of both sexes diagnosed with schizophrenia using the Structured Clinical Interview for DSM-5 Disorders, Clinician Version (SCID-5-CV) [28], aged 18–65 years, was recruited from February 2018 to February 2020; 40 ‘inpatients’ lived in four transitional halfway houses (i.e., psychiatric hostels) for at least 3 months and 40 ‘outpatients’ lived with their families and were followed up in two general hospital outpatient clinics. All patients had to be on antipsychotic medication, free of relapse, and with no need for psychiatric hospitalization during the last 3 months. Exclusion criteria for inpatients’ admission into the hostels were intellectual disability, history of alcoholism or drug use in the last 6 months, and current severe medical conditions (e.g., neurological degenerative diseases, brain lesions). These exclusion criteria were also applied in recruiting both inpatients and outpatients. Clinicodemographic characteristics were recorded for all patients.

In addition, all twenty-two nurses working in the four halfway houses and caring for the 40 inpatients and 56 parents of the 40 outpatients participated in the study as raters of their EE toward patients. An additional exclusion criterion for raters was a lifetime diagnosis of a psychotic disorder.

No eligible patient or nurse denied participation. Four eligible parents refused to participate, although their spouse agreed. These were not significantly different in sex, age, and education from the 56 parents who agreed to participate.

Both patients and raters were ensured of the anonymity and confidentiality of all data requested and provided written informed consent before participation in the study. The research protocol followed the principles of the Helsinki Declaration and was approved by the Research Ethics Committees of all mental health facilities involved.

### 2.2. Measurements

Patients of both groups went through the following evaluations:

- Brief Psychiatric Rating Scale (BPRS): BPRS originally included 16 interviewer-rated items assessing the intensity of symptoms of schizophrenia [29]. The most commonly used 18-item version with the addition of excitement and disorientation in 1966 has a five-factor structure, including Thinking disorder, Withdrawal, Anxiety/Depression, Hostility/Suspicion, and Activity factors [30].
- Perceived Criticism (PC): The PC instrument was introduced to measure perceived criticism in a sample of depressed patients and their spouses [31] but it has since been used with several other populations, including patients with schizophrenia [32,33]. It consists of only one self-rated question rated on a 10-point Likert scale: “How critical do you feel hostel nurses/your parents have been of you overall in the last month?”.

Finally, both staff nurses and outpatients’ parents participated in the following procedure:

- Five Minutes Speech Sample (FMSS): The FMSS [34] is a tool for measuring EE. In comparison to the CFI, the standard assessment tool of EE, the FMSS is easier to use, needs far less time to administer, and requires shorter training of the interviewer. It can also be used even when the researcher does not know the patient very well. Each rater (i.e., care provider or family member) is asked to talk continuously for 5 min about each patient (in his/her absence) and the interview is audiotaped. All recorded 5 min interviews are then scored according to specific rules based on the assessment of the following: (a) the initial statement in terms of content and voice tone, (b) the quality of the patient–rater relationship, (c) the number of negative or positive comments, and (d) the display or report of specific behaviors during the interview (see Supplementary Methods for details on scoring). Every 5 min speech sample is eventually characterized as high, borderline, or low on Criticism and EOI; combined classifications may also arise (e.g., ‘high critical’, ‘high EOI’, ‘high critical + EOI’). FMSS interviews were scored by a trained author (S.D.) and an acceptable inter-rater agreement with another trained author (P.F.) was recorded in 20 interviews (criticism, EOI kappa = 0.89; critical comments ICC = 0.91; positive attitude statements ICC = 0.90).

### 2.3. Statistical Analysis

The distribution of all variables was explored with descriptive statistics. Normality was checked with the Shapiro–Wilk test and graphically with histograms and QQ plots. The reliability (internal consistency) of the returned questionnaires was evaluated with Cronbach’s alpha. Differences in the characteristics of participants (i.e., patients and nurses or parents) and EE outcomes between the two groups were evaluated with Chi-square, Fisher’s exact, t-test, or Mann–Whitney tests, as appropriate.

Four EE outcomes were evaluated. Apart from our two primary categorical EE outcomes (i.e., FMSS-Criticism and FMSS-EOI), the number of FMSS critical comments and the number of FMSS positive attitude statements were also used as secondary criticism and EOI outcomes, respectively. As frequencies of low FMSS-Criticism/EOI categories were very small, they were lumped together with borderline FMSS-Criticism/EOI categories in downstream univariate and multivariate analyses.

EE ratings derived from FMSS interviews were a multilevel/hierarchical dataset of observations. In particular, each outpatient was rated only by his/her own parent(s), hence parents’ ratings were ‘nested’ within outpatients, while each inpatient received ratings from various nurses and each nurse rated several patients (‘crossed levels’). As each nurse’s ratings as well as ratings for each patient were non-independent/correlated, mixed-effects models were justified. Mixed models incorporate random effects accommodating clusters of correlated ratings apart from fixed effects [35]. As none of our EE outcomes were normally distributed, linear mixed models were not appropriate. Therefore, generalized linear mixed models (GLMMs) were selected, which model an appropriate transformation of the outcome depending on its distribution [35]. For binary outcomes, i.e., high vs. borderline/low FMSS-Criticism and FMSS-EOI, binary logistic GLMMs were fitted, producing an odds ratio (OR) for each predictor. For count outcomes, that is, those taking only non-negative integer values, i.e., the number of FMSS critical comments and FMSS positive attitude statements, negative binomial and Poisson GLMMs were fitted, respectively, producing an incidence rate ratio (IRR) for each predictor. GLMMs with crossed effects were initially fitted but did not converge on most occasions. Therefore, simpler GLMMs with ratings nested within patients were finally fitted. First, univariate GLMMs were assessed to investigate the unadjusted effect of the patient group and patient-related characteristics (including BPRS subscales) on raters’ (nurses’ or parents’) EE in the total sample. The adjusted effect of the patient group on EE outcomes was then estimated in multivariate GLMMs after adding appropriate confounders. As confounders of the effect of the patient group on EE, we considered all patient-related characteristics that were significantly associated with the patient group or with EE outcomes in univariate GLMMs [36]. In the last step, adjustment was extended to add any rater-related characteristics present in both groups, i.e., raters’ age, sex, and education.

Finally, using multilevel structural equation models in MPlus [37], we checked whether patient- or rater-related confounders mediated the effect of the patient group on EE outcomes. In mediation analyses, the total/unadjusted effect of the patient group on an EE outcome (also assessed in univariate GLMMs) was decomposed into a direct/adjusted effect (also estimated in multivariate GLMMs) and the indirect effects of all mediators included in the model. The 95% CI of the indirect effects was calculated with a Monte Carlo (MC) simulator. Significant indirect effects informed us on which patient and caregiver characteristics, when different between settings, also drove EE differences.

Statistical analysis was conducted in STATA MP v17 and MPlus 7.1. Given the exploratory nature of the study, the level of statistical significance was set to *p* < 0.05 (two-tailed). However, to avoid Type I error inflation from multiple tests (22 EE predictors for each of the 4 EE outcomes), an adjusted *p*-value cut-off of 0.00057 was applied to identify the most robust effects.

Approximate power calculations before recruitment were performed using a web-based R application developed for estimating power in linear and generalized linear mixed models [38]. For our participants nested within two equal-size groups, we assumed an effect size of Cohen’s *d* = 0.5 for group differences (i.e., moderate by Cohen’s rule of thumb [39]), corresponding to an OR = 2.48 [40] or an IRR = 1.86 [41], participants’ and raters’ random variance components of 0.3 each, error variance of 0.4, and no rater by group interaction. We also hypothesized a mean of 4 nurses’ ratings per inpatient and 1.5 parents’ ratings per outpatient, resulting in an overall mean of 2.75 ratings per patient. Based on these assumptions, the required total sample size to obtain a power of 0.8 was 65.6 patients. We finally targeted 80 participants (40 inpatients, 40 outpatients) to compensate for potential dropouts and provide additional power for smaller effect sizes of other predictors.

Post hoc empirical power calculations were performed with Monte Carlo simulations using the ‘mixedpower’ R package [42], in which we simulated 500 datasets using our fitted univariate GLMMs for EE outcomes, the final sample size, and data structure but varying the effect size (OR or IRR) of the predictor. Empirical power was estimated as the proportion of significant simulations to all simulations.

## 3. Results

### 3.1. Sample Descriptives, Univariate Comparisons, and Correlations

#### 3.1.1. Patients (Inpatients–Outpatients) and Raters (Nurses–Parents)

Demographic characteristics of the patients and raters by group are presented in Table 1. Inpatients were older (*p* < 0.001) and less well educated (*p* = 0.002) than outpatients, and they had a longer disease duration (*p* = 0.025) as well as more hospitalizations (*p* = 0.012). Nurses were significantly younger (*p* < 0.001) and better educated (*p* = 0.007) than parents. Seven (12.5%) parents had a lifetime psychiatric history of depression.

Cronbach’s *α* for BPRS total was 0.783 and ranged between 0.703 and 0.728 for BPRS subscales. Outpatients had significantly higher scores in BPRS Withdrawal (*p* = 0.015), BPRS Total (*p* = 0.027), and PC (*p* < 0.001) than inpatients (Table 1). However, both patient groups were overall remitted/mildly ill [43].

#### 3.1.2. EE Ratings

Each of the 22 nurses was involved in 1–12 EE ratings (FMSS interviews); each of the 40 inpatients was rated by 2–5 (mean 3.88) nurses. A total of 155 ratings were performed by nurses on inpatients. All 56 parents rated their offspring; each of the 40 outpatients was rated by 1 or both (mean 1.4) parents. Overall, each patient was rated by a mean of 2.64 caregivers.

A comparison of primary EE outcomes between groups is displayed in Table 2. FMSS components, based on which EE outcomes are scored, are presented in Table 3 for the two groups. Parents did not significantly differ from nurses in FMSS-Criticism, critical and dissatisfaction comments, or FMSS-EOI. Yet, they made significantly more positive attitude statements (*p* = 0.003) and exhibited self-sacrificing or overprotective behavior (*p* = 0.005) and intense emotional displays during the interview (*p* < 0.001) significantly more often than nurses. However, nurses made significantly more positive comments (*p* < 0.001) and reported excessive detail about the past significantly more often (*p* = 0.011). Finally, a marginally significant (*p* = 0.049) difference in the distribution of combined EE categories but not binary EE categories (high vs. low EE) was detected between groups.

### 3.2. Univariate Patient-Related Predictors of EE Outcomes in the Total Sample

First, univariate GLMMs with patient-related predictors were fitted for all EE outcomes in the total sample (Table 4). As previously shown (Table 2 and Table 3), the effect of the patient group was significant only for FMSS positive attitude statements (IRR = 1.69, *p* = 0.019). Higher criticism in any criticism outcome (i.e., FMSS-Criticism or FMSS critical comments) was significantly predicted by patients being employed vs. unemployed, higher BPRS Withdrawal, and higher PC. Higher EOI in any EOI outcome (i.e., FMSS-EOI or FMSS positive attitude statements) was significantly predicted by outpatient group, higher age, female sex, unemployed or retired status, lower BPRS Thinking disorder, Withdrawal, Hostility/Suspicion, Activity, and Total. Only the effect of BPRS Total on FMSS-EOI survived adjustment for multiple tests.

### 3.3. Adjusting for Patient-Related Confounders Only

#### 3.3.1. Effect of Patient Group on EE Outcomes after Adjusting for Patient-Related Confounders Only

In EE multivariate GLMMs, after adjusting for patient-related confounders, that is, all variables significantly associated with the patient group (Table 1) or with EE outcomes in univariate GLMMs (Table 4), higher criticism in any criticism outcome was significantly predicted by higher PC but not the patient group, while higher EOI in any EOI outcome was significantly predicted by outpatient group, higher age, female sex (trend *p* = 0.05), unemployment, previous hospitalizations, lower BPRS Withdrawal, and lower PC (Table 5). The effect of patient groups on FMSS positive attitude statements was amplified after adjustment (direct effect IRR = 2.61, *p* = 0.0003) and remained significant after adjustment for multiple tests. Furthermore, a patient group effect on FMSS-EOI was uncovered (direct effect OR = 2.79, *p* = 0.027), with outpatients being more likely to have a high FMSS-EOI. Non-significant adjusted group effects were recorded for FMSS-Criticism (OR = 0.71, *p* = 0.577) and FMSS critical comments (IRR = 0.52, *p* = 0.105).

#### 3.3.2. Patient-Related Mediators of the Effect of Patient Group on EE Outcomes

**Figure 1:**
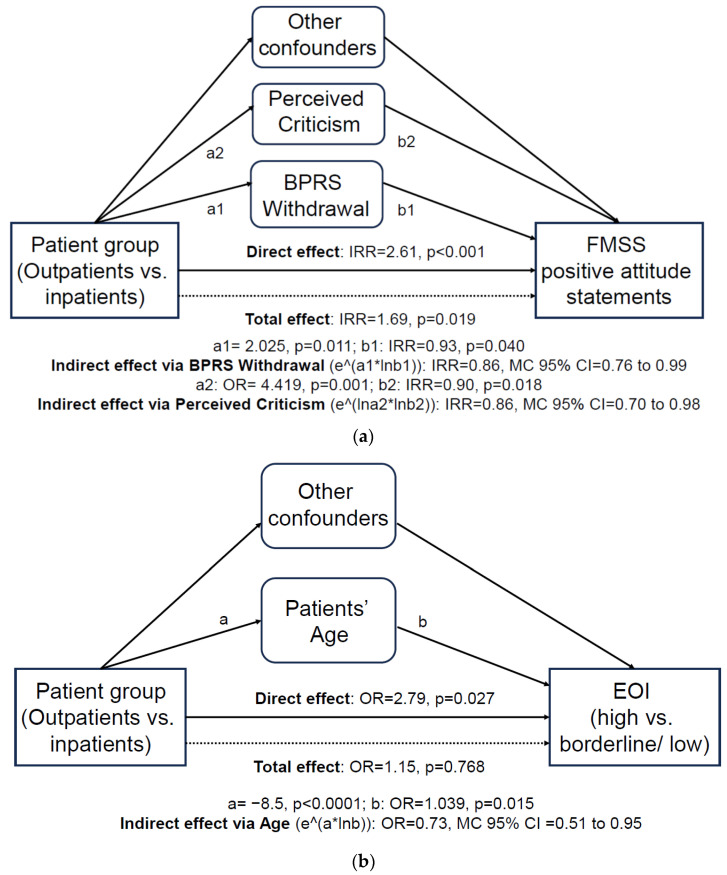
Schematic representation of patient-related characteristics significantly mediating group effects on EOI outcomes in multilevel structural equation models: (**a**) FMSS positive attitude statements; (**b**) FMSS-EOI. BPRS, Brief Psychiatric Rating Scale; EOI, Emotional Overinvolvement; FMSS, Five Minutes Speech Sample.

Multilevel structural equation models (MSEMs) in MPlus 7.1 tested the mediatory role of patient-related confounders in the effect of patient groups (outpatients vs. inpatients) on EE outcomes. Significant ‘negative’ (<1) indirect effects of groups on FMSS positive attitude statements via BPRS Withdrawal (indirect effect IRR = 0.86, MC 95% CI = 0.76 to 0.99) and PC (indirect effect IRR = 0.86, MC 95% CI = 0.70 to 0.98) were detected (Figure 1a). Furthermore, a significant ‘negative’ (<1) indirect effect of groups on FMSS-EOI via age (indirect effect OR = 0.73, MC 95% CI = 0.51 to 0.95) was detected (Figure 1b). These significant ‘negative’ (i.e., opposite direction) indirect effects, when ‘added’ to corresponding aforementioned significant ‘positive’ direct/adjusted effects (see Section 3.3.1 and Table 4) along with non-significant indirect effects of other patient-related confounders, respectively, resulted in a weaker ‘positive’, yet significant, total/unadjusted group effect on FMSS positive attitude statements (IRR = 1.69, *p* = 0.019) and a ‘positive’ non-significant total/unadjusted group effect on FMSS-EOI (OR = 1.15, *p* = 0.768; see Table 4). Finally, we detected a significant ‘positive’ (>1) indirect effect of group on FMSS-Criticism via PC (indirect effect OR = 1.39, MC 95% CI = 1.05 to 2.11), which, when ‘added’ to our ‘negative’ (<1) non-significant direct/adjusted effect (OR = 0.71, *p* = 0.577; see Section 3.3.1 and Table 5) along with non-significant indirect effects of other patient-related confounders, resulted in a ‘positive’ non-significant total/unadjusted effect of group on FMSS-Criticism (OR = 1.40, *p* = 0.499; see Table 4).

### 3.4. Adjusting for Both Patient- and Rater-Related Confounders

#### 3.4.1. Effect of Patient Group on EE Outcomes after Adjusting for Patient- and Rater-Related Confounders

In the last step, adjustment in EE multivariate GLMMs was extended to include both patient-related confounders and any rater-related predictors present in both groups, i.e., age, sex, and education (Table 1). Interestingly, in these final ‘combined’ models, adjusted group effects on both EOI outcomes became non-significant, while adjusted group effects on both criticism outcomes now turned significant (FMSS-Criticism OR = 0.08, *p* = 0.027; FMSS critical comments IRR = 0.16, *p* = 0.035), with inpatients scoring higher (Table 5). Higher criticism in any criticism outcome was also significantly predicted by patients’ current employment, higher BPRS Withdrawal, and higher PC, while higher EOI in any EOI outcome was significantly predicted by higher patient age (trend *p* = 0.052), female sex, unemployment, lower BPRS Withdrawal, and lower PC. Furthermore, higher rater age was significantly associated with higher criticism; rater female sex was significantly associated with both higher criticism and EOI. In the final ‘combined’ models, no effect survived our adjusted *p*-value cut-off.

#### 3.4.2. Patient- and Rater-Related Mediators of the Effect of Patient Group on EE outcomes

MSEMs in the final models tested the mediatory role of both patient- and rater-related confounders in the effect of patient groups on EE outcomes. In MSEMs for rater-related confounders, we detected a significant ‘positive’ (>1) indirect effect of groups on FMSS-Criticism via rater age (indirect effect OR = 6.86, MC 95% CI = 1.70 to 28.47) and significant ‘negative’ (<1) indirect effects of group on FMSS critical comments (indirect effect IRR = 0.49, MC 95% CI = 0.24 to 0.85) and FMSS-EOI (indirect effect OR = 0.73, MC 95% CI = 0.48 to 0.99) via rater female sex. In MSEMs for patient-related confounders, we detected a significant ‘positive’ (>1) indirect effect of group on FMSS-Criticism via PC (indirect effect OR = 1.45, MC 95% CI = 1.07 to 2.28) and a significant ‘negative’ (<1) indirect effect of group on FMSS positive attitude statements via PC (indirect effect IRR = 0.85, MC 95% CI = 0.70 to 0.98).

**Figure 2:**
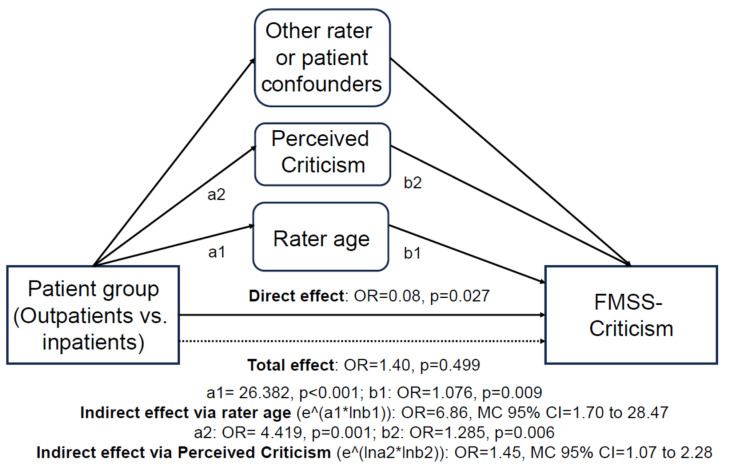
Schematic representation of patient- and rater-related characteristics significantly mediating group effects on FMSS-Criticism in multilevel structural equations models. FMSS, Five Minutes Speech Sample.

Therefore, for example, our significant ‘positive’ indirect effects of groups on FMSS-Criticism via rater age and via PC, when ‘added’ to our significant ‘negative’ direct effect (OR = 0.08, *p* = 0.027; see Section 3.4.1. and Table 5) along with non-significant indirect effects of other patient- and rater-related confounders, resulted in our non-significant ‘positive’ total/unadjusted effect of groups on FMSS-Criticism (OR = 1.40, *p* = 0.499; see Table 4) (Figure 2).

### 3.5. Post Hoc Empirical Power Calculations

In post hoc empirical power calculations with the ‘mixedpower’ R package, patient group and other predictors were tested with EE outcomes in univariate GLMMs. The estimated power to detect an at least moderate effect (OR = 2.48, corresponding to *d* = 0.5 [40]) of groups on FMSS-Criticism and FMSS-EOI was 0.364 and 0.37, respectively, while the estimated power to detect an at least moderate effect (IRR = 1.86, corresponding to *d* = 0.5 [41]) of groups on FMSS positive attitude statements was 0.814. Regarding other predictors, the estimated power to detect, for example, an at least small effect of PC (OR = 1.44, corresponding to *d* = 0.2 [40]) on FMSS-Criticism and an at least small effect of BPRS Withdrawal (OR = 0.696, corresponding to *d* = −0.2) on FMSS-EOI was 0.964 and 0.99, respectively. Therefore, our univariate models were underpowered to detect group effects on FMSS-Criticism and FMSS-EOI but adequately powered to detect group effects on FMSS positive attitude statements as well as effects of other predictors, such as PC and BPRS Withdrawal, on FMSS-Criticism and FMSS-EOI, respectively.

## 4. Discussion

This study adds to a large body of literature on EE and its correlates in families of patients with schizophrenia as well as a smaller, more recent body of literature in staff–patient settings, such as supported residential facilities in the community. Our study is novel in simultaneously recording and directly comparing EE between parents and professional caregivers after adjusting for potential confounders. We first controlled only for patient-related confounders and then added basic caregiver-related demographics available in both groups. Finally, we searched for mediatory effects of patient- and caregiver-related confounders to investigate pathways underlying EE differences between the two settings.

Most previous studies used patient–caregiver dyads after arbitrarily selecting one ‘primary’ caregiver for each patient to simplify statistical analyses, yet unavoidably introducing bias. Instead, we have allowed each patient to be rated by 1–2 parents or 2–5 nurses while each nurse rated 1–12 patients. In the very few studies using such nested or crossed-level data collection, non-independence of observations was either ignored [13], disproved with simple tests [44,45], or recognized yet without proceeding to multilevel modeling in analyses [24]. Therefore, another key strength of this study was our recruitment scheme (‘one patient by many raters, one rater for many patients’), which necessitated the use of mixed models in statistical analysis but provided more valid and less biased EE ratings.

A median high EE rate of 54% (range 23–77%) [46] and mean rates of 51% (range 23–76%) for high EE, 46.9% (range 25–94%) for high criticism, and 36% (range 12–72%) for high EOI [8] have been reported in meta-analyses of familial EE in schizophrenia. Therefore, the rate of high EE in parents (Table 2) is at the upper end of meta-analytical reports but rates of high criticism and EOI are less than 1 S.D. higher than the means. On the other hand, high EE rates are typically lower than 40% in staff–patient studies, with negligible rates of high EOI [22], though with a few exceptions [47,48]. Therefore, the rate of high EE in nurses (Table 2) was unexpectedly high and indeed slightly higher than in parents. Yet, the rates of high criticism and high EOI were nominally lower in nurses, who still made nominally more critical, dissatisfaction, and positive comments than parents, contributing to the balanced picture of the two groups. The rates of high criticism were, expectedly, larger than the rates of high EOI in both settings. Cultural variation [49], especially in EOI [50], individual characteristics of families and hostels, and the author’s scoring style might provide an explanation for these inflated EE rates.

Various patient-related predictors of criticism and EOI outcomes were identified in univariate models in the total sample (Table 4). Higher criticism was significantly predicted in our study by patients being employed vs. unemployed, higher BPRS Withdrawal (i.e., more severe negative symptoms), and higher PC. In the literature, higher criticism was associated with negative symptoms or reduced social functioning in hostels [10,44] but not in families [32,51,52] and with higher PC in both hostels [12,53] and families [54,55]. On the other hand, higher criticism was previously associated with unemployment and poor job-related skills (rather than being currently employed) in both hostels [56] and families [57]. Higher EOI was significantly predicted in our study by higher age, female sex, unemployed or retired status, and less severe psychotic symptoms (i.e., lower BPRS Thinking disorder, Withdrawal, Hostility/Suspicion, Activity, and Total). In the literature, higher EOI was associated with higher patient age in hostels [10] but lower age in families [58,59]. Furthermore, higher EOI in families was previously associated with patients’ unemployment [59], higher depression/anxiety, and lower aggression [60]. The remaining findings have not been previously reported.

Regarding caregiver-related demographics (i.e., age, sex, and education), age was associated with higher criticism and female sex with both higher criticism and EOI in the final ‘combined’ multivariate models. In the literature, higher criticism in hostels was associated with higher nurse age [56] and lower education [10]. Moreover, higher EOI in families was more often associated with parents’ female sex [59,60,61,62] but also with male sex [58]. Concludingly, previous literature on both patient- and caregiver-related EE predictors is highly heterogeneous, since different sample characteristics can confound associations, and studies often use different EE measures and scoring algorithms. For example, FMSS and CFI have modest concordance rates, with FMSS considered less sensitive in identifying high-EE individuals [6].

Before adjustment, our primary EE outcomes, FMSS-Criticism and FMSS-EOI, did not significantly differ between groups; yet, parents reported significantly more FMSS positive attitude statements, a secondary EOI outcome (total/unadjusted effects; Table 4). Therefore, if the two groups had been included in two separate studies and these were indirectly compared (without adjusting for confounders), one could at best report only a minor excess of EOI in parents. However, the two groups had significant differences in patients’ clinicodemographic characteristics, psychopathology, and PC, as shown in Table 1, which might partly explain or confound EE differences between settings. The significant indirect effects of patients’ age, BPRS Withdrawal, and PC on EOI outcomes were ‘inconsistent’ [63], that is, they ‘favored’ inpatients and were of the opposite direction to total and, a fortiori, direct effects ‘favoring’ outpatients. That is, both EOI outcomes were significantly higher in outpatients/parents after adjusting for patient-related confounders, an effect that was robust to correction for multiple tests (direct/adjusted effects; Table 5). However, this effect was attenuated in unadjusted comparisons because outpatients’ lower age and higher BPRS Withdrawal and PC were compatible with lower EOI. Therefore, our findings based on direct adjusted comparison between the two settings corroborate previous indirect, yet consistent, comparisons pointing to higher EOI in parents than staff nurses [22].

Compared to criticism, high EOI is more weakly associated with poor outcomes in meta-analyses [8] and inconsistently across cultures [50]. An explanation of this finding is that the effect of EOI on outcomes is moderated by the inter-related, often coexistent but less well-studied dimension of warmth [50,64], which mediates the protective aspects of the family environment through mutual support in kin relationships [9]. Therefore, higher EOI in parents suggests more intrusive and overprotective behaviors but also more concern, interest, empathy, and emotional investment in their offspring, while lower EOI in nurses may suggest a lack of interest and disengagement from patients [5].

The two groups also significantly differed in caregivers’ age and education. Families were characterized by age and education gaps between outpatients and their parents, since outpatients were younger and better educated than their parents, which were reversed between inpatients and nurses in hostels. After additionally adjusting for caregiver-related demographics, particularly caregiver age and sex with significant mediatory effects, nurses significantly outweighed parents on both criticism outcomes while differences in EOI outcomes became non-significant. In other words, if both patient and caregiver characteristics were assumed similar across settings, parents and nurses would be similar in EOI outcomes, although nurses are professional and not natural caregivers, while nurses would be more critical, probably due to their professional status. However, when adjusting only for patient-related confounders, these effects were overridden because, for example, parents’ older age was compatible with higher criticism. In all multivariate models, a significant indirect effect of patients’ PC on FMSS-Criticism was also found. Noticeably, one cannot safely infer the direction of causality between caregiver criticism and patients’ PC as circular causation is highly probable.

Finally, a cautionary note should be made on statistical adjustment. Although the two groups were artificially matched on several characteristics with statistical procedures, differences in both patient and caregiver profiles between the two settings may often exist in real life [65]. For example, patients in hostels are often more chronic, of lower socioeconomic and educational status, and usually required to have achieved symptomatic remission to be accepted, while professional caregivers are often younger and more educated than parents. Therefore, statistical adjustment mainly serves to investigate pathways (mediatory effects) underlying EE differences between the two settings rather than forcing them to be identical. We showed that EE differences across settings may be attributed to differences in patient-related characteristics and basic caregiver demographics.

Before adjustment, EE rates were equally high in both settings. After adjustment, we have confirmed a deficit of EOI (and probably warmth) in halfway houses compared to families but also showed a potential excess of criticism depending on caregivers’ characteristics. Therefore, psychoeducation for both staff [66] and family members [67,68] is warranted to improve their caregiving capacity, by mitigating criticism and EOI while sparing their positive feelings (warmth and concern) and increasing their degree of engagement with patients. Our findings regarding EE predictors (Table 4 and Table 5) might, hence, help customize the objectives or target groups of psychoeducational interventions. Higher criticism arose from older, female caregivers toward autonomous (employed) patients with higher PC or toward patients with higher negative symptoms. Lower EOI and disengagement were displayed by male caregivers toward younger, male, employed patients with higher PC and toward patients with more severe psychopathology, esp. negative symptoms and agitation/aggression. Therefore, psychoeducation should aim to provide education about schizophrenia, negative symptoms, and aggression and modify their causal attributions to personal, internal, and controllable factors [12,13], in order to curb criticism, particularly in older caregivers, help them better cope with patients’ aggression and perceived criticism, and help increase concern for patients with negative symptoms by motivating behavioral and lifestyle interventions [69] or by encouraging caregivers’ engagement with vocational and social skills training programs [70].

Limitations of our study include the following: (a) Both groups had a relatively small sample size and the number of ratings performed was quite different among them (56 in outpatients vs. 155 in inpatients). Post hoc empirical power calculations in univariate models showed inadequate power to detect group effects on FMSS-Criticism and FMSS-EOI but adequate power for group effects on FMSS positive attitude statements as well as effects of other patient-related predictors, such as PC and BPRS Withdrawal. Yet, in multivariate models, significant group effects on FMSS-Criticism and FMSS-EOI could in fact be detected after adjusting for confounders. (b) Our study did not include various patient (e.g., duration of untreated psychosis, cognitive functioning, and social cognition) and caregiver characteristics (e.g., burden, distress, causal attributions/illness perceptions, coping strategies, and personality profile) that might also explain EE differences between groups [10,12,17,51,52,57,62]. Further research is warranted on directly comparing supported housing facilities and families on caregivers’ EE using adequately powered samples and adjusting for additional patient and caregiver characteristics in order to shed more light on pathways underlying EE differences between these two settings.

## 5. Conclusions

EE toward patients with schizophrenia is typically reported to be lower in halfway houses than in families, with negligible rates of high EOI. However, comparisons have been indirect, potentially suffering from biases. This is the first study simultaneously including both patients with schizophrenia living in halfway houses or with their families and directly comparing EE across groups, adjusting for patient- and caregiver-related confounders. Before adjustment, criticism and EOI rates were equally high in both settings. After adjusting for patient-related confounders only, EOI was significantly higher in parents, corroborating previous indirect comparisons, while criticism did not significantly differ between groups. However, after also adjusting for caregiver demographics, criticism was significantly higher in nurses, while differences in EOI became non-significant. Patients’ age, negative symptoms, and perceived criticism and caregivers’ age and sex significantly mediated group differences in EE outcomes. Therefore, our study highlights pathways potentially underlying EE differences between halfway houses and families; future comparative studies should investigate additional patient and caregiver characteristics as mediators of EE differences across settings. Finally, our findings underscore the importance of psychoeducational interventions for both families and staff to increase their caregiving capacity for patients with schizophrenia.

## Figures and Tables

**Table 1:** Description of patient sample (40 inpatients, 40 outpatients) and basic demographics for raters (22 nurses, 56 parents).

	Inpatients N = 40	Outpatients N = 40	*p*-Value
Sex (Male)	27 (67.5%)	22 (55.0%)	0.251 ^a^
Age (years)	48.6 *±* 9.3	40.1 *±* 7.8	**<0.001** ^c^
Family status			1.000 ^b^
Single	34 (85.0%)	33 (82.5%)	
Married	1 (2.5%)	2 (5.0%)	
Divorced/Widowed	5 (12.5%)	5 (12.5%)	
Education			**0.002** ^a^
Primary/High School	33 (82.5%)	20 (50.0%)	
University or higher	7 (17.5%)	20 (50.0%)	
Employment			0.762 ^b^
Employed	7 (17.5%)	10 (25.0%)	
Unemployed	30 (75.0%)	27 (67.5%)	
Retired	3 (7.5%)	3 (7.5%)	
Smoking	28 (70.0%)	24 (60.0%)	0.348 ^a^
History of violent behavior	14 (35.0%)	10 (25.0%)	0.329 ^a^
History of suicide attempts	3 (7.5%)	3 (7.5%)	1.000 ^b^
Duration of disease (years)	18.2 *±* 10.9	13.4 *±* 7.2	**0.025** ^c^
No. of hospitalizations	2 (2–4)	2 (1–3)	**0.012** ^d^
BPRS Thinking disorder	5 (4–7.5)	6.5 (5–9.5)	0.190 ^d^
BPRS Withdrawal	6 (4–8)	8.5 (6–11.5)	**0.015** ^d^
BPRS Anxiety/Depression	7.5 (6–9.5)	9 (6.5–12)	0.162 ^d^
BPRS Hostility/Suspicion	4 (3–5.5)	4 (3–6.5)	0.964 ^d^
BPRS Activity	3 (3–4)	3 (3–4.5)	0.345 ^d^
BPRS Total	28 (22.5–37)	33.5 (28.5–40.5)	**0.027** ^d^
Perceived Criticism	3.7 *±* 2.4	5.80 *±* 2.66	**<** **0.001 ^c^**
	**Nurses (N = 22)**	**Parents (N = 56** **)**	
Sex (Male)	6 (27.3%)	24 (42.9%)	0.203 ^a^
Age (years)	40.0 *±* 7.2	68.0 *±* 8.6	**<0.001** ^c^
Education			**0.007** ^a^
Primary/High School	8 (36.4%)	39 (69.6%)	
University or higher	14 (63.6%)	17 (30.4%)	

N (%), median (IQR), or mean *±* SD are presented.; Chi-square ^a^, Fisher’s exact ^b^, *t*-test ^c^, or Mann–Whitney ^d^ tests were used as appropriate.; BPRS, Brief Psychiatric Rating Scale; Bold, *p* < 0.05.

**Table 2:** Comparison of expressed emotion (EE) outcomes between nurses/inpatients and parents/outpatients.

Five Minutes Speech Sample (FMSS) Outcomes	Nurses	Parents	*p*-Value
Criticism			0.310 ^a†^
High	84 (54.2%)	33 (58.9%)	
Borderline	65 (41.9%)	23 (41.1%)	
Low	6 (3.9%)	0 (0.0%)	
Emotional Overinvolvement (EOI)			0.940 ^b††^
High	72 (46.5%)	27 (48.2%)	
Borderline	65 (41.9%)	22 (39.3%)	
Low	18 (11.6%)	7 (12.5%)	
EE Categories (n = 7)			**0.049** ^a§^
High critical	54 (34.8%)	17 (30.4%)	
High EOI	42 (27.1%)	11 (19.6%)	
High critical + EOI	30 (19.4%)	16 (28.6%)	
Borderline critical	1 (0.6%)	4 (7.1%)	
Borderline EOI	0 (0.0%)	0 (0.0%)	
Borderline critical + EOI	28 (18.1%)	8 (14.3%)	
Low critical + EOI	0 (0.0%)	0 (0.0%)	
EE Categories (n = 2)			0.659 ^b^
High EE	126 (81.3%)	44 (78.6%)	
Low EE	29 (18.7%)	12 (21.4%)	

Inpatient data came from 155 nurses’ FMSS ratings; each of the 22 nurses was involved in 1–12 ratings; each of the 40 inpatients was rated by 2–5 nurses. Data for outpatients came from 56 parents rating their offspring; each of the 40 outpatients was rated by 1 or both parents.; N (%) are presented.; ^a^ Fisher’s exact; ^b^ Chi-square; ^†^ *p* = 0.541 for high vs. borderline/low FMSS-Criticism; ^††^ *p* = 0.821 for high vs. borderline/low FMSS-EOI; ^§^ *p* = 0.405 for four FMSS categories (high critical, high EOI, high critical + EOI, borderline/low critical + EOI).; Bold, *p* < 0.05.

**Table 3:** Comparison of FMSS components between nurses/inpatients and parents/outpatients.

		Inpatients (N = 155 Ratings)	Outpatients (N = 56 Ratings)	*p*-Value
**FMSS-Criticism**				
Critical comments		1.55 *±* 2.40	1.12 *±* 1.94	0.446 ^a^
Critical comments ≥ 1		59 (38.1%)	20 (35.7%)	0.755 ^b^
Dissatisfaction comments		3.54 *±* 2.47	3.00 *±* 2.00	0.241 ^a^
Dissatisfaction comments ≥1		138 (89.0%)	48 (85.7%)	0.510 ^b^
Initial statement	Negative	38 (24.5%)	15 (26.8%)	0.217 ^b^
	Neutral	64 (41.3%)	16 (28.6%)	
	Positive	53 (34.2%)	25 (44.6%)	
Quality of relationship	Negative	59 (38.1%)	21 (37.5%)	0.717 ^b^
	Neutral	16 (10.3%)	8 (14.3%)	
	Positive	80 (51.6%)	27 (48.2%)	
**FMSS-EOI**				
Positive attitude statements		0.55 *±* 0.82	0.89 *±* 0.87	**0.003 ^a^**
Positive attitude statements ≥ 1		59 (38.1%)	35 (62.5%)	**0.002 ^b^**
Positive comments		7.10 *±* 3.53	4.61 *±* 3.33	**<0.001 ^a^**
Positive comments ≥ 5		115 (74.2%)	26 (46.4%)	**<0.001 ^b^**
Self-sacrificing or overprotective behavior		0 (0%)	4 (7.1%)	**0.005 ^c^**
Intense emotional display during interview		1 (0.6%)	7 (12.5%)	**<0.001 ^c^**
Excessive detail about the past		53 (34.2%)	9 (16.1%)	**0.011 ^b^**

N (%) or mean *±* S.D. are presented; ^a^ Mann–Whitney test; ^b^ Chi-square; ^c^ Fisher’s exact test; EOI, Emotional Overinvolvement; FMSS, Five Minutes Speech Sample; Bold, *p* < 0.05.

**Table 4:** Univariate patient-related predictors (*p* < 0.1) of expressed emotion outcomes in the total sample.

	FMSS—Criticism (OR, *p*)	FMSS Critical Comments (IRR, *p*)	FMSS—EOI (OR, *p*)	FMSS Positive Attitude Statements (IRR, *p*)
**Patient Predictors**	Logit, Patients	NB, Patients	Logit, Patients	Poisson, Patients
Group (outpatients vs. inpatients)	1.40, 0.499	0.72, 0.319	1.15, 0.768	**1.69** **, 0.019**
Sex (female vs. male)				**1.73** **, 0.014**
Age (years)			**1.06, 0.011**	
Family status (ever married vs. single)				
Education (university or higher vs. lower)			2.38, 0.092	
Employment (Ref. employed)		Unemployed: **0.40, 0.006** Pensioner: -	Unemployed: **4.46, 0.006** Pensioner: **7.25, 0.025**	
Smoking				
Disease duration (years)				
No. of previous hospitalizations				
History of violent behavior				
History of suicide attempts				
BPRS Thinking disorder			**0.87** **, 0.025**	
BPRS Withdrawal		**1.09** **, 0.032**	**0.84, 0.004**	
BPRS Anxiety/Depression				
BPRS Hostility/Suspicion			**0.77, 0.026**	
BPRS Activity			**0.73** **, 0.027**	
BPRS Total			0.93, 0.00048 *	0.98, 0.090
Perceived Criticism	**1.26, 0.013**		0.86, 0.090	

Except for group, only predictors with *p* < 0.1 are presented; BPRS, Brief Psychiatric Rating Scale; EOI, Emotional Overinvolvement; FMSS, Five Minutes Speech Sample; Logit = Binary Logistic Generalized Linear Mixed Model; NB = Negative Binomial Generalized Linear Mixed Model; Patients = ratings were nested within patients; Poisson = Poisson Generalized Linear Mixed Model; OR > 1 and IRR > 1 denote positive associations; Bold, *p* < 0.05; * *p* < 0.00057 (adjusted *p*-value cut-off).

**Table 5:** Effect of patient group in multivariate models of expressed emotion outcomes in the total sample, adjusting for patient-related confounders only (upper line of each cell) or both patient- and rater-related confounders (lower line of each cell).

	FMSS—Criticism (OR, *p*)	FMSS Critical Comments (IRR, *p*)	FMSS—EOI (OR, *p*)	FMSS Positive Attitude Statements (IRR, *p*)
**Patient predictors**	Logit, Patients	NB, Patients	Logit, Patients	Poisson, Patients
Group (outpatients vs. inpatients)	0.71, 0.577 **0.08, 0.027**	0.52, 0.105 **0.16, 0.035**	**2.79, 0.027** 1.49, 0.644	2.61, 0.00031 * 1.40, 0.486
Sex (female vs. male)				1.48, 0.050 **1.48, 0.049**
Age (years)	0.99, 0.775 0.95, 0.199	1.01, 0.503 0.99, 0.680	**1.07, 0.006** 1.05, 0.052	1.00, 0.920 0.99, 0.489
Family status (ever married vs. single)				
Education (university or higher vs. lower)	1.07, 0.895 1.26, 0.681	0.88, 0.741 0.93, 0.837	1.35, 0.453 1.52, 0.317	0.87, 0.519 0.91, 0.649
Employment (Ref. employed)		Unemployed: 0.55, 0.087 **0.49, 0.046** Pensioner: 1.74, 0.329 1.68, 0.345	Unemployed: **4.09, 0.005** **4.37, 0.005** Pensioner: 3.37, 0.109 3.57, 0.108	
Smoking				
Disease duration (years)	1.00, 0.980 1.01, 0.659	0.98, 0.385 0.99, 0.710	0.96, 0.053 0.96, 0.131	1.01, 0.420 1.01, 0.293
No. of previous hospitalizations	1.04, 0.698 1.01, 0.881	1.06, 0.254 1.05, 0.381	**1.15, 0.044** 1.14, 0.076	1.02, 0.525 1.02, 0.637
History of violent behavior				
History of suicide attempts				
BPRS Thinking disorder			0.94, 0.303 0.93, 0.268	
BPRS Withdrawal	1.06, 0.373 1.09, 0.199	1.07, 0.108 **1.10, 0.023**	**0.87, 0.020** **0.87, 0.027**	**0.93, 0.032** 0.94, 0.058
BPRS Anxiety/Depression				
BPRS Hostility/Suspicion			0.86, 0.131 0.86, 0.152	
BPRS Activity			0.99, 0.914 1.04, 0.781	
Perceived Criticism	**1.06, 0.023** **1.27, 0.023**	**1.15, 0.024** **1.18, 0.010**	1.01, 0.939 1.02, 0.766	**0.91, 0.022** **0.90, 0.018**
**Rater predictors**				
Age (years)	- **1.08, 0.021**	- 1.04, 0.108	- 1.03, 0.268	- 1.02, 0.156
Sex (female vs. male)	- 2.14, 0.076	- **2.45, 0.008**	- **3.23, 0.003**	- 1.37, 0.156
Education (university or higher vs. lower)	- 0.99, 0.973	- 1.22, 0.502	- 1.17, 0.644	- 0.91, 0.592

In each cell, upper line = patient-related confounders only, lower line = both patient- and rater (nurse or parent)-related confounders; BPRS, Brief Psychiatric Rating Scale; EOI, Emotional Overinvolvement; FMSS, Five Minutes Speech Sample; Logit = Binary Logistic Generalized Linear Mixed Model; NB = Negative Binomial Generalized Linear Mixed Model; Poisson = Poisson Generalized Linear Mixed Model; Patients = ratings were nested within patients; OR > 1 and IRR > 1 denote positive associations; Bold, *p* < 0.05; * *p* < 0.00057 (adjusted *p*-value cut-off).

## Data Availability

The study dataset is available upon reasonable request.

## Supplementary Materials

The following supporting information can be downloaded at https://www.mdpi.com/article/10.3390/healthcare12030375/s1, Supplementary Methods.
