# Supplementary material for: Family in Crisis: Do Halfway Houses Perform Better Than Families with Expressed Emotion toward Patients with Schizophrenia? A Direct Adjusted Comparison
Source: Healthcare (Basel). 2024 Feb 1;12(3):375. doi: 10.3390/healthcare12030375 (PMC10855104; doi:10.3390/healthcare12030375)
Supplement: Supplementary file 1 [file healthcare-12-00375-s001.zip › healthcare-2838582-supplementary.pdf]

Supplementary Methods

FMSS scoring

Criticism and Emotional Overinvolvement (EOI), the two dimensions of EE, are rated based on conditions grouped in four categories: initial statement, quality of relationship, criticism, and EOI. The first three are used to rate Criticism and the last to rate EOI. Criticism and EOI are finally coded as high, borderline and low. Borderline can also be grouped with low to provide a dichotomous rating (high vs borderline/low).

High Criticism is coded if the rater (a) makes a negative initial statement about the patient; the initial statement can be rated as negative, neutral or positive; or (b) describes the relationship with the patient as negative; statements about the quality of the relationship are individually rated as weakly or strongly negative (-1 or -2) or positive (+1 or +2) and then summed up giving the overall relationship rating (negative, neutral or positive); or (c) makes at least one critical (i.e. strongly disapproving) comment, rated on the basis of content and voice tone. **Borderline Criticism** is coded if the rater only makes dissatisfaction comments.

High EOI is coded if the rater (a) reports or displays overprotective or self-sacrificing behavior or lack of objectivity towards the patient; or (b) exhibits intense emotional display during the interview; or (c) any two of the following are true: (1) describes the past or a minor characteristic of the patient in excessive detail, (2) makes at least one statement of positive attitude towards the patient, (3) makes five or more positive comments about the patient. **Borderline EOI** is coded if only one of the aforementioned (c) conditions is true.

The final seven EE categories arise from combining Criticism and EOI categories: 'high critical', 'high EOI', 'high critical+EOI', 'borderline critical', 'borderline EOI', 'borderline critical+EOI', 'low critical+EOI' (or 'low EE'). If borderline is grouped with low, one will have four categories: 'high critical', 'high EOI', 'high critical+EOI', 'borderline/low critical+EOI'. Finally, one can have two major categories of 'high EE' (critical and/or EOI) and 'borderline/low EE' (critical and/or EOI).
